# Sparse canonical methods for biological data integration: application to a cross-platform study

**DOI:** 10.1186/1471-2105-10-34

**Published:** 2009-01-26

**Authors:** Kim-Anh Lê Cao, Pascal GP Martin, Christèle Robert-Granié, Philippe Besse

**Affiliations:** 1 Station d'Amélioration Génétique des Animaux UR 631, Institut National de la Recherche Agronomique, F-31326 Castanet, France; 2 Institut de Mathéematiques, Université de Toulouse et CNRS (UMR 5219), F-31062 Toulouse, France; 3 Laboratoire de Pharmacologie et Toxicologie UR 66, Institut National de la Recherche Agronomique, F-31931 Toulouse, France

## Abstract

**Background:**

In the context of systems biology, few sparse approaches have been proposed so far to integrate several data sets. It is however an important and fundamental issue that will be widely encountered in post genomic studies, when simultaneously analyzing transcriptomics, proteomics and metabolomics data using different platforms, so as to understand the mutual interactions between the different data sets. In this high dimensional setting, variable selection is crucial to give interpretable results. We focus on a sparse Partial Least Squares approach (sPLS) to handle two-block data sets, where the relationship between the two types of variables is known to be symmetric. Sparse PLS has been developed either for a regression or a canonical correlation framework and includes a built-in procedure to select variables while integrating data. To illustrate the canonical mode approach, we analyzed the NCI60 data sets, where two different platforms (cDNA and Affymetrix chips) were used to study the transcriptome of sixty cancer cell lines.

**Results:**

We compare the results obtained with two other sparse or related canonical correlation approaches: CCA with Elastic Net penalization (CCA-EN) and Co-Inertia Analysis (CIA). The latter does not include a built-in procedure for variable selection and requires a two-step analysis. We stress the lack of statistical criteria to evaluate canonical correlation methods, which makes biological interpretation absolutely necessary to compare the different gene selections. We also propose comprehensive graphical representations of both samples and variables to facilitate the interpretation of the results.

**Conclusion:**

sPLS and CCA-EN selected highly relevant genes and complementary findings from the two data sets, which enabled a detailed understanding of the molecular characteristics of several groups of cell lines. These two approaches were found to bring similar results, although they highlighted the same phenomenons with a different priority. They outperformed CIA that tended to select redundant information.

## Background

In systems biology, it is particularly important to simultaneously analyze different types of data sets, specifically if the different kind of biological variables are measured on the same samples. Such an analysis enables a real understanding on the relationships between these different types of variables, for example when analyzing transcriptomics, proteomics or metabolomics data using different platforms. Few approaches exists to deal with these high throughput data sets. The application of linear multivariate models such as Partial Least Squares regression (PLS, [1]) and Canonical Correlation Analysis (CCA, [2]), are often limited by the size of the data set (ill-posed problems, CCA), the noisy and the multicollinearity characteristics of the data (CCA), but also the lack of interpretability (PLS). However, these approaches still remain extremely interesting for integrating data sets. First, because they allow for the compression of the data into 2 to 3 dimensions for a more powerful and global view. And second, because their resulting components and loading vectors capture dominant and latent properties of the studied process. They may hence provide a better understanding of the underlying biological systems, for example by revealing groups of samples that were previously unknown or uncertain. PLS is an algorithmic approach that has often been criticized for its lack of theoretical justifications. Much work still needs to be done to demonstrate all statistical properties of the PLS (see for example [3,4] who recently addressed some theoretical developments of the PLS). Nevertheless, this computational and exploratory approach is extremely popular thanks to its efficiency.

Recent integrative biological studies applied Principal Component Analysis, or PLS [5,6], but for a regression framework, where prior biological knowledge indicates which type of omic data is expected to explain the other type (for example transcripts and metabolites). Here, we specifically focus on a canonical correlation framework, when there is either no assumption on the relationship between the two sets of variables (exploratory approach), or when a reciprocal relationship between the two sets is expected (*e.g*. cross platform comparisons). Our interests lie in integrating these two high dimensional data sets and perform variable selection simultaneously. Some sparse associated integrative approaches have recently been developed to include a built-in selection procedure. They adapt lasso penalty [7] or combine lasso and ridge penalties (Elastic Net, [8]) for feature selection in integration studies.

In this study, we propose to apply a sparse canonical approach called "sparse PLS" (sPLS) for the integration of high throughput data sets. Methodological aspects and evaluation of sPLS in a regression framework were presented in [9]. This novel computational method provides variable selection of two-block data sets in a one step procedure, while integrating variables of two types.

When applying canonical correlation-based methods, most validation criteria used in a regression context are not statistically meaningful. Instead, the biological relevancy of the results should be evaluated during the validation process. In this context, we compare sparse PLS with two other canonical approaches: penalized CCA adapted with Elastic Net (CCA-EN [10]), which is a sparse method that was applied to relate gene expression with gene copy numbers in human gliomas, and Co-Inertia Analysis (CIA, [11]) that was first developed for ecological data, and then for canonical high-throughput biological studies [12]. This latter approach does not include feature selection, which has to be performed in a two-step procedure. This comparative study has two aims. First to better understand the main differences between each of these approaches and to identify which method would be appropriate to answer the biological question, second to highlight how each method is able to reveal the underlying biological processes inherent to the data. This type of comparative analysis renders biological interpretation mandatory to strengthen the statistical hypothesis, especially when there is a lack of statistical criteria to assess the validity of the results. We first recall some canonical correlation-based methods among which the two sparse methods, sPLS and CCA-EN will be compared with CIA on the NCI60 cell lines data set. We propose to use appropriate graphical representations to discuss the results. The different gene lists are assessed, first with some statistical criteria, and then with a detailed biological interpretation. Finally, we discuss the pros and cons of each approach before concluding.

### Canonical correlation-based methods

We focus on two-block data matrices denoted *X*(*n *× *p*) and *Y *(*n *× *q*), where the *p *variables *x*^*j *^and *q *variables *y*^*k *^are of two types and measured on the same samples or individuals *n*, for *j *= 1 ... *p *and *k *= 1 ... *q*. Prior biological knowledge on these data allows us to settle into a canonical framework, *i.e*. there exists a reciprocal relationship between the *X *variables and the *Y *variables. In the case of high throughput biological data, the large number of variables may affect the exploratory method, due to numerical issues (as it is the case for example with CCA), or lack of interpretability (PLS).

We next recall three types of multivariate methods (CCA, PLS, CIA). For CCA and PLS, we describe the associated sparse approaches that were proposed, either to select variables from each set or to deal with the ill-posed problem commonly encountered in high dimensional data sets.

#### CCA

Canonical Correlation Analysis [2] studies the relationship between two sets of data. The CCA *n*-dimensional score vectors (*Xa*_*h*_, *Yb*_*h*_) come in pairs to solve the objective function:

$$\begin{array}{cc} \arg\underset{{a^{\prime }}_{h}a_{h}=1,{b^{\prime }}_{h}b_{h}=1}{\max}cor(Xa_{h},Yb_{h}), & h=1...H, \end{array}$$

where the *p*- and *q*-dimensional vectors *a*_*h *_and *b*_*h *_are called canonical factors, or loading vectors, and *h *is the CCA chosen dimension. As *cor*(*Xa*_*h*_, *Yb*_*h*_) = *cov*(*Xa*_*h*_, *Yb*_*h*_)/$\sqrt{var(Xa_{h})}\sqrt{var(Yb_{h})}$, the aim of CCA is to simultaneously maximize *cov*(*Xa*_*h*_, *Yb*_*h*_) and minimize the variances of *Xa*_*h *_and *Yb*_*h*_. It is known that the CCA loadings are not directly interpretable [13]. It is however very instructive to interpret these components by calculating the correlation between the original data set *X *and {*a*_1_, ..., *a*_*H*_} and similarly between *Y *and {*b*_1_, ..., *b*_*H*_}, to project variables onto correlation circles. Easier interpretable graphics are then obtained, as shown in the R package cca [14].

In the *p *+ *q *>> *n *framework, CCA suffers from high dimensionality as it requires the computation of the inverse of two covariance matrices *XX' *and *YY ' *that are singular. This implies numerical difficulties, since the canonical correlation coefficients are not uniquely defined. One solution proposed by [15] was to ntroduce *l*_2 _penalties in a ridge CCA (rCCA) on the covariance matrices, so as to make them invertible. rCCA was recently applied to genomic data [16], but was not adapted in our study as it does not perform feature selection. We focused instead of another variant called CCA with Elastic Net penalization (see below).

#### PLS

Partial Least Squares regression [1] is based on the simultaneous decomposition of *X *and *Y *into latent variables and associated loading vectors. The latent variables methods (*e.g*. PLS, Principal Component Regression) assume that the studied system is driven by a small number of *n*-dimensional vectors called latent variables. These latter may correspond to some biological underlying phenomenons which are related to the study [17]. Like CCA, the PLS latent variables are linear combinations of the variables, but the objective function differs as it is based on the maximization of the covariance:

$$\begin{array}{cc} \arg\underset{{a^{\prime }}_{h}a_{h}=1,{b^{\prime }}_{h}b_{h}=1}{\max}cov(X_{h-1}a_{h},Yb_{h}), & h=1...H, \end{array}$$

where *X*_*h*-1 _is the residual (deflated) *X *matrix for each PLS dimension *h*. We denote *ξ*_*h *_and *ω*_*h *_the *n*-dimensional vectors called "latent variables" which are associated to each loading vector *a*_*h *_and *b*_*h*_. In contrary to CCA, the loading vectors (*a*_*h*_, *b*_*h*_) are interpretable and can give information about how the *x*^*j *^and *y*^*k *^variables combine to explain the relationships between *X *and *Y*. Furthermore, the PLS latent variables (*ξ*_*h*_, *ω*_*h*_) indicate the similarities or dissimilarities between the individuals, related to the loading vectors.

Many PLS algorithms exist, not only for different shapes of data (SIMPLS, [18], PLS1 and PLS2 [1], PLS-SVD [19]) but also for different aims (predictive, like PLS2, or modelling, like PLS-mode A, see [10,20,21]). In this study we especially focus on a modelling aim ("*canonical mode*") between the two data sets, by deflating *X *and *Y *in a symmetric way (see Additional file 1).

#### CCA-EN

[10] proposed a sparse penalized variant of CCA using Elastic Net [8,22] for a canonical framework. To do so, the authors used the PLS-mode A formulation [20,21] to introduce penalties. Note that Elastic Net is well adapted to this particular context. It combines the advantages of the ridge regression, that penalizes the covariance matrices *XX' *and *YY*' which become non singular, and the lasso [7] that allows variable selection, in a one step procedure. However, when *p *+ *q *is very large, the resolution of the optimization problem requires intensive computations, and [8,10] proposed instead to perform a univariate thresholding, that leaves only the lasso estimates to compute (see Additional file 1).

#### sparse PLS

[9] proposed a sparse PLS approach (sPLS) based on a PLS-SVD variant, so as to penalize both loading vectors *a*_*h *_and *b*_*h *_simultaneously.

For any matrix M (*p *× *q*) of rank *r*, the SVD of M is given by:

*M *= *A*Δ*B*',

where the columns of *A *(*p *× *r*) and *B*(*q *× *r*) are orthonormal and contain the eigenvectors of *MM*' and *M*'*M*, Δ (*r *× *r*) is a diagonal matrix of the squared eigenvalues of *MM*' or *M*'*M*. Now if *M *= *X*'*Y*, then the column vectors of *A *(resp. *B*) correspond to the loading vectors of the PLS *a*_*h *_(resp. *b*_*h*_). Sparsity can then be introduced by iteratively penalizing *a*_*h *_and *b*_*h *_with a soft-thresholding penalization, as [23] proposed for a sparse PCA using SVD computation. Both regression and canonical deflation modes were proposed for sPLS [9]. In this paper, we will focus on the canonical mode only (see Additional file 1 for more details of the algorithm). The regression mode has already been discussed in [9] with a thorough biological interpretation of the results.

#### CIA

Co-Inertia analysis (CIA) was first introduced by [11] in the context of ecological data, before being applied to high throughput biological data by [12]. CIA is suitable for a canonical framework, as it is adapted for a symmetric analysis. It involves analyzing each data set separately either with principal component analyses, or with correspondence analyses, such that the covariance between the two new sets of projected scores vectors (that maximize either the projected variability or inertia) is maximal. This results in two sets of axes, where the first pair of axes are maximally co-variant, and are orthogonal to the next pair [24]. CIA does not propose a built-in variable selection, but we can perform instead a two-step procedure by ordering the weight vector (loadings) for each CIA dimension and by selecting the top variables.

#### Differences between the approaches

These three canonical based approaches, CCA-EN, sPLS and CIA profoundly differ in their construction, and hence their aims. On the one hand, CCA-EN looks for canonical variate pairs (*Xa*_*h*_, *Yb*_*h*_), such that a penalized version of the canonical correlation is maximized. This explains why a non monotonic decreasing trend in the canonical correlation can sometimes be obtained [10]. On the other hand, sPLS (canonical mode) and CIA aim at maximizing the covariance between the scores vectors, so that there is a strong symmetric relationship between both sets. However, here CIA is based on the construction of two Correspondence Analyses, whereas sPLS is based on a PLS analysis.

#### Parameters tuning

In CCA-EN, the authors proposed to tune the penalty parameters for each dimension, such that the canonical correlation *cor*(*Xa*_*h*_, *Yb*_*h*_) is maximized. In practice, they showed that the correlation did not change much when more variables were added in the selection. Therefore, an appropriate way of tuning the parameters would be to choose instead the degree of sparsity (*i.e*. the number of variables to select), as previously proposed for sparse PCA by [22,23]-see the elasticnet R package for example, and hence to rely on the biologists needs. Thus, depending on the aim of the study (focus on few genes or on groups of genes such as whole pathways) and on the ability to perform follow-up studies, the size of the selection can be adapted. When focusing on groups of genes (*e.g*. pathways, transcription factor targets, variables involved in the same biological process), we believe that the selection should be large enough to avoid missing specific functions or annotations. The same strategy will be used for sPLS (see also [9] where the issue of tuning sPLS parameters is addressed). No other parameters than the number of selected variables is needed in CIA either.

#### Outputs

Graphical representations are crucial to help interpreting the results. We therefore propose to homogenize all outputs to enable their comparison.

Samples are represented with the scores or latent variable vectors, in a superimposed manner, as proposed in the R package ade4 [25], first to show how samples are clustered based on their biological characteristics, and second to measure if both data sets strongly agree according to the applied approach. In these graphical representations, each sample is indicated using an arrow. The start of the arrow indicates the location of the sample in the *X *data set in one plot, and the tip of the arrow the location of the sample in the *Y *data set in the other plot. Thus, short (long) arrows indicate if both data sets strongly agree (disagree) between the two data sets.

Variables are represented on correlation circles, as previously proposed by [14]. Correlations between the original data sets and the score or latent variable vectors are computed so that highly correlated variables cluster together in the resulting graphics. Only the selected variables in each dimension are represented. This type of graphic not only allows for the identification of interactions between the two types of variables, but also for identifying the relationship between variable clusters and associated sample clusters. Note that for large variable selections, the use of interactive plotting, color codes or representations limited to user-selected variables may be required to simplify the outputs.

### Cross-platform study

#### Data sets and relevance for a canonical correlation analysis

We chose to compare the three canonical correlation-based methods (CCA-EN, CIA and sPLS) for their ability to highlight the relationships between two gene expression data sets both obtained on a panel of 60 cell lines (NCI60) from the National Cancer Institute (NCI). This panel consists of human tumor cell lines derived from patients with leukaemia (LE), melanomas (ME) and cancers of ovarian (OV), breast (BR), prostate (PR), lung (LU), renal (RE), colon (CO) and central nervous system (CNS) origin. The NCI60 is used by the Developmental Therapeutics Program (DTP) of the NCI to screen thousands of chemical compounds for growth inhibition activity and it has been extensively characterized at the DNA, mRNA, protein and functional levels. The data sets considered here have been generated using Affymetrix [26,27] or spotted cDNA [28] platforms. These data sets are highly relevant to an analysis in a canonical framework since 1) there is some degree of overlap between the genes measured by the two platforms, but also a large degree of complementarity through the screening of different gene sets representing common pathways or biological functions [12] and 2) they play fully symmetric roles, as opposed to a regression framework where one data set is explained by the other. We assume that the data sets are correctly normalized, as described below.

#### The Ross Data set

[28] used spotted cDNA microarrays containing 9,703 human cDNAs to profile each of the 60 cell line in the NCI60 panel [28]. Here, we used a subset of 1,375 genes that has been selected using both non-specific and specific filters described in [29]. In particular, genes with more than 15% of missing values were removed and the remaining missing values were imputed by *k*-nearest neighbours [12]. The pre-processed data set containing log ratio values is available in [12].

#### The Staunton Data set

Hu6800 Affymetrix microarrays containing 7,129 probe sets were used to screen each of the 60 cell lines in another study [26,27]. Pre-processing steps are described in [27] and [12]. They include 1) replacing average difference values less than 100 by an expression value of 100, 2) eliminating genes whose expression was invariant across all 60 cell lines and 3) selecting the subset of genes displaying a minimum change in expression across all 60 cell lines of at least 500 average difference units. The final analyzed data set contained the average difference values for 1,517 probe sets, and is available in [12].

#### Application of the three sparse canonical correlation-based methods

We applied CCA-EN, CIA and sPLS to the Ross (*X*) and Staunton (*Y*) data sets. For each dimension *h*, *h *= 1 ... 3, we selected 100 genes from each data set. The number of dimensions was arbitrarily chosen, as when *H *≥ 4, the analysis of the results becomes difficult given the high number of graphical outputs. Indeed, for higher dimensions, the cell lines did not cluster by their tissue of origin, which made their interpretation more difficult. The size of the selection (100) was judged small enough to allow for the identification of individual relevant genes and large enough to reveal gene groups belonging to the same functional category or pathway.

## Results and Discussion

We apply the three canonical correlation-based approaches to the NCI60 data set and assess the results in two different ways. First we examine some statistical criteria, then we provide a biological interpretation of the results from each method, using graphical representations along with database mining.

### How to assess the results?

Canonical correlation-based methods are statistically difficult to assess. Firstly, because they do not fit into a regression/prediction framework, meaning that the prediction error cannot be estimated using cross-validation to evaluate the quality of the model. Secondly, because in many two-block biological studies, the number of samples *n *is very small compared to the number of variables *p *+ *q*. This makes any statistical criteria difficult to compute or estimate. This is why graphical outputs are important to help analyze the results (see for example [12,20]).

When working with biological data, a new way of assessing the results should be to strongly rely on biological interpretation. Indeed, our aim is to show that each approach is applicable and to assess whether they answer the biological question. We therefore propose to base most of our comparative study on the biological interpretation of the results by using appropriate graphical representations of the samples and the selected variables.

### Link between two-block data sets

#### Variance explained by each component

[20] proposed to estimate the variance explained in each data set *X *and *Y *in relation to the "opposite" component score or latent variables (*ω*_1_, ..., *ω*_*H*_) and (*ξ*_1_, ..., *ξ*_*H*_), where *ξ*_*h *_= *Xa*_*h *_and *ω*_*h *_= *Yb*_*h *_in all approaches. The redundancy criterion *Rd*, or part of explained variance, is computed as follows:

$$Rd(X;\omega_{1},...,\omega_{H})=\frac{1}{p}\sum_{h=1}^{H}\sum_{j=1}^{p}cor^{2}(x^{j},\omega_{h}),$$

$$Rd(Y;\xi_{1},...,\xi_{H})=\frac{1}{q}\sum_{h=1}^{H}\sum_{k=1}^{q}cor^{2}(y^{k},\xi_{h}).$$

Similarly, one can compute the variance explained in each component in relation with its associated data set:

$$Rd(X;\xi_{1},...,\xi_{H})=\frac{1}{p}\sum_{h=1}^{H}\sum_{j=1}^{p}cor^{2}(x^{j},\xi_{h}),$$

$$Rd(Y;\omega_{1},...,\omega_{H})=\frac{1}{q}\sum_{h=1}^{H}\sum_{k=1}^{q}cor^{2}(y^{k},\omega_{h}).$$

**Figure 1:**
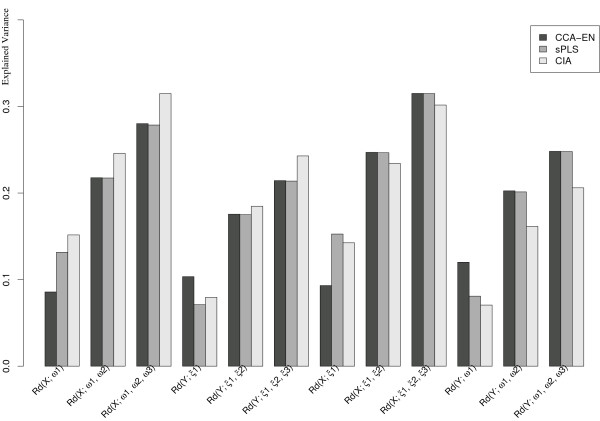
**Rd**. Cumulative explained variance (Rd criterion) of each data set in relation to its component score (CCA-EN, CIA) or latent variable (sPLS).

**Figure 2:**
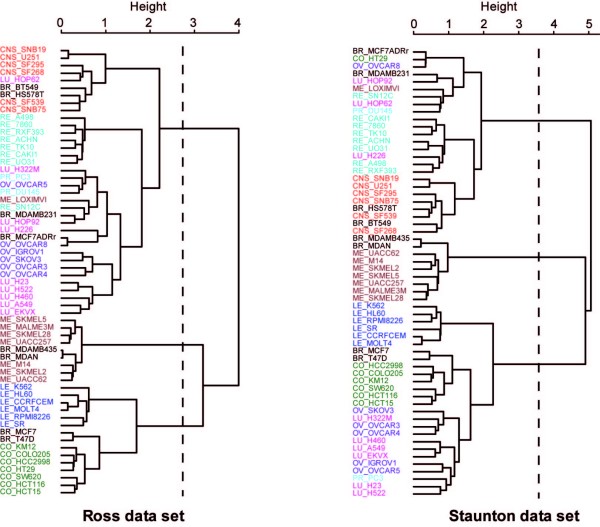
**Hierarchical clustering of the two data sets using all expression profiles**. Hierarchical clustering of the cell lines with Ward method and correlation distance using the expression profiles from the Ross (left) and Staunton (right) data sets. The tissues of origin of the cell lines are coded as BR = Breast, CNS = Central Nervous System, CO = Colon, LE = Leukaemia, ME = Melanoma, LU = Lung, OV = Ovarian, PR = Prostate, RE = Renal. The Ward method maximizes the between-cluster inertia and minimizes the within-cluster inertia for each step of the clustering algorithm. Height represents the loss of between-cluster inertia for each clustering step. Dashed lines cut the dendrograms to highlight the three main clusters.

Figure 1 displays the *Rd *criterion for *h *= 1 ... 3 for each set of components (*ξ*_1_, *ξ*_2_, *ξ*_3_) (*ω*_1_, *ω*_2_, *ω*_3_) and for each approach. While there seems to be a great difference in the first dimension between CCA and the other methods, the components in dimensions 2 and 3 explain the same amount of variance in both *X *and *Y *for CCA-EN and sPLS. This suggests a strong similarity between these two approaches at this stage. However, CIA differs from these two methods. The components computed from the "opposite" set explain more variance than CCA/sPLS, and less in their respective set. Overall, we can observe that more information seems to be present in the *X *(Ross) rather than in the *Y *(Staunton) data set. Indeed, similarly to [12], we noticed that a hierarchical clustering of the samples from the Ross data set allows a better clustering of the cell lines based on their tissue of origin than from the Staunton data set (Figure 2).

#### Correlations between each component

The canonical correlations between the pair of score vectors or latent variables were very high (>0.93) for any approach and in any dimension (see Table 1). This confirms our hypothesis regarding the canonical aim of each method. The non monotonic decreasing trend of the canonical correlations in CCA-EN is not what can be expected from a CCA variant. This fact was also pointed out by [10] as the optimization criterion in CCA-EN differs from ordinary CCA. However, the computations of the *Rd *criterion (Figure 1) seem to indicate that the cumulative variance explained by the latent variables increases with *h*. sPLS and CIA also highlight very strongly correlated components, as their aim is to maximize the covariance. This suggests that the associated loading vectors may also bring related information regarding the variables (genes) from both data sets. The maximal canonical correlation (≃ 0.97) is obtained on the first dimension for CCA-EN, and surprisingly, only on the second dimension for CIA and sPLS. In the next sections, we show that, in fact, CCA-EN and sPLS permute their components between the first and second dimensions.

**Table 1:** Correlations. Correlations between the score vectors (CCA-EN, CIA) or between latent variables (sPLS) for each dimension.

	CCA-EN	CIA	sPLS
*cor*(*ξ*_1_, *ω*_1_)	0.967	0.935	0.938
*cor*(*ξ*_2_, *ω*_2_)	0.937	0.967	0.964
*cor*(*ξ*_3_, *ω*_3_)	0.953	0.955	0.944

### Interpretation of the observed cell line clusters

#### Graphical representation of the samples

**Figure 3:**
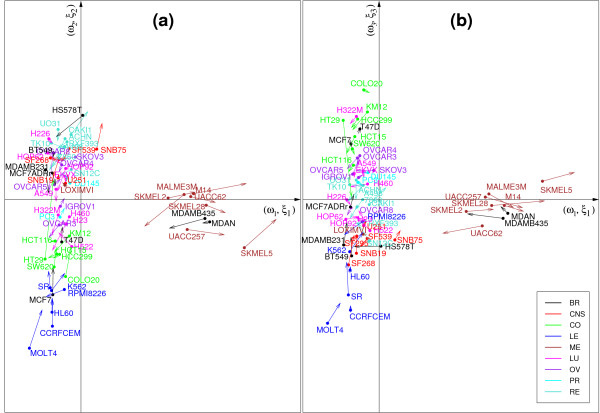
**Graphical representations of the samples using CCA-EN**. Graphical representations of the cell lines by plotting the component scores from CCA-EN from dimension 1 and 2 **(a) **or 1 and 3 **(b)**. The component scores computed on each data set are displayed in a superimposed manner, where the start of the arrow shows the location of the Ross samples, and the tip the Staunton samples. Short arrows indicate if both data sets strongly agree. The colors indicate the tissues of origin of the cell lines with BR = Breast, CNS = Central Nervous System, CO = Colon, LE = Leukaemia, ME = Melanoma, LU = Lung, OV = Ovarian, PR = Prostate, RE = Renal.

**Figure 4:**
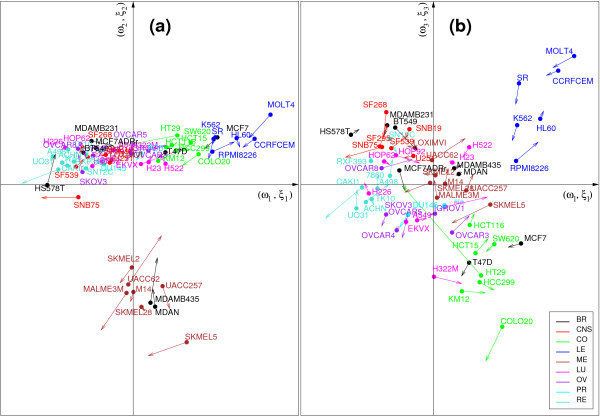
**Graphical representations of the samples using sPLS**. Graphical representations of the cell lines by plotting the latent variable vectors from sPLS from dimension 1 and 2 **(a) **or 1 and 3 **(b)**. The latent variable vectors computed on each data set are displayed in a superimposed manner, where the start of the arrow shows the location of the Ross samples, and the tip the Staunton samples. Short arrows indicate if both data sets strongly agree. The colors indicate the tissues of origin of the cell lines with BR = Breast, CNS = Central Nervous System, CO = Colon, LE = Leukaemia, ME = Melanoma, NS = Lung, OV = Ovarian, PR = Prostate, RE = Renal.

Figures 3 and 4 display the graphical representations of the samples in dimension 1 and 2 **(a)**, or 1 and 3 **(b) **for CCA-EN (Figure 3) and sPLS (Figure 4). CIA showed similar patterns to sPLS and to those presented in [12]. All graphics show that both data sets are strongly related (short arrows), but the components differ, depending on the applied method. In dimension 1, the pair (*ξ*_1_, *ω*_1_) tends to separate the melanoma cell lines from the other cell lines in CCA-EN (Figure 3(a)), whereas sPLS and CIA tend to separate the LE and CO cell lines on one side from the RE and CNS cell lines on the other side (Figure 4(a)). As previously proposed by [12], we interpreted this latter clustering as the separation of cell lines with *epithelial *characteristics (mainly LE and CO) from those with *mesenchymal *characteristics (in particular RE and CNS). Epithelial cells generally form layers by making junctions between them and interacting with the extracellular matrix (ECM), whereas mesenchymal cells are able to migrate through the ECM and are found in the connective tissues. In dimension 2, we observe the opposite tendency: the CCA-EN score vectors (*ξ*_2_, *ω*_2_) separates the cell lines with epithelial characteristics from the cell lines with mesenchymal characteristics (Figure 3(a)), while the sPLS or CIA pair (*ξ*_2_, *ω*_2_) separates the melanoma samples from the other samples (Figure 4(a), not shown for CIA). Finally, in dimension 3 all three methods separate the LE from the CO cell lines.

#### Hierarchical clustering of the samples

To further understand this difference between the methods, we separately performed hierarchical clustering of the 60 cell lines for each data set (Figure 2). The main clusters that we identified corresponded to the three groups of cell lines which were previously highlighted by the three methods (Figures 3 and 4):

1) cell lines with epithelial characteristics (mainly LE and CO),

2) cell lines with mesenchymal characteristics (in particular RE and CNS) and

3) ME cell lines which systematically clustered with MDA_N and MDA_MB435. These latter cell lines are indeed melanoma metastases derived from a patient diagnosed with breast cancer. As previously reported [12,28,29], ME cell lines (including MDA_N and MDA_MB435) form a compact and homogeneous cluster which is strictly identical between the two data sets. Only the LOXIMVI cell line, which lacks melanin and several typical markers of melanoma cells [30] did not cluster with all ME cell lines (Figure 2). CCA-EN first focused on separating ME *vs*. the other cell lines, a cluster that seems consistent in both data sets. In contrast, sPLS and CIA first focused on the separation between epithelial *vs*. mesenchymal cell lines characteristics, even though most OV and LU cell lines clustered either with the mesenchymal-like cell lines (Ross data set) or with the epithelial-like cell lines (Staunton data set) in Figure 2. This illustrates an important difference between CCA-EN and sPLS/CIA: by maximizing the correlation, CCA-EN first focuses on the most conserved clusters between the two data sets. To evaluate this hypothesis, we artificially reduced the consistency in the ME clustering by permuting some of the labels of the melanoma cell lines with other randomly selected cell lines in one of the data set. The resulting graphics in CCA-EN happened to be similar to those obtained for sPLS and CIA in the absence of permutation (Figure 3(a)), separating epithelial-like *vs*. mesenchymal-like cell lines on the first dimension. By contrast, sPLS and CIA graphics remained the same after the permutations. Thus it seems that the maximal correlation can only be obtained through a high consistency of the clusterings between the two data sets. However, CCA-EN may be more strongly affected by the few samples that would not cluster similarly in the two data sets, that is, by a low consistency between the two data sets.

### Interpretation of the observed genes clusters

#### Graphical representation of the genes

**Figure 5:**
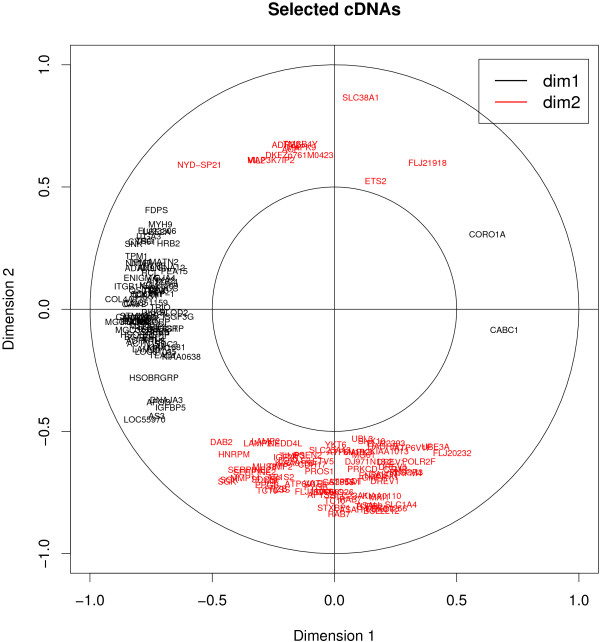
**Graphical representations of the variables selected by sPLS, Ross data set**. Example of graphical representation of the genes selected on the first two sPLS dimensions. The coordinates of each gene are obtained by computing the correlation between the latent variable vectors (*ξ*_1_, *ξ*_2_) and the original Ross data set. The selected cDNAs are then projected onto correlation circles where highly correlated cDNAs cluster together. These graphics help identifying correlated genes between the two platforms (by superimposing graphics from Figures 5 and 6). They also allow for the association between the gene clusters and a type of tumor cell lines by combining the information contained in Figure 4. The labels of the cDNAs can be plotted interactively in R to facilitate their identification. Subsets of the selected genes may also be displayed alone to focus on specific, user-defined, gene groups.

**Figure 6:**
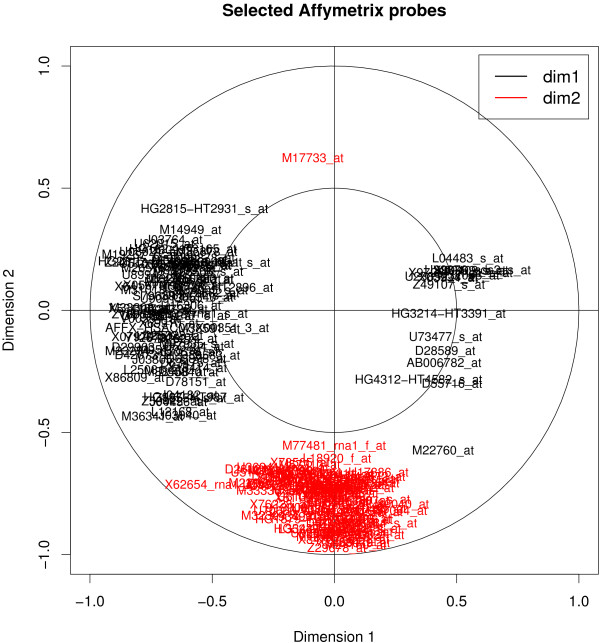
**Graphical representations of the variables selected by sPLS, Staunton data set**. Example of graphical representation of the genes selected on the first two sPLS dimensions. The coordinates of each gene are obtained by computing the correlation between the latent variable vectors (*ω*_1_, *ω*_2_) and the original Staunton data set. The selected Affymetrix probes are then projected onto correlation circles where highly correlated probes cluster together. These graphics help identifying correlated genes between the two platforms (by superimposing graphics from Figures 5 and 6). They also allow for the association between the gene clusters and a type of tumor cell lines by combining the information contained in Figure 4. The labels of the Affymetrix probes can be plotted interactively in R to facilitate their identification. Subsets of the selected genes may also be displayed alone to focus on specific, user-defined, gene groups.

We computed the correlations between the original data sets and the scores vectors or latent variables (*ξ*_1_, *ξ*_2_, *ξ*_3_) and (*ω*_1_, *ω*_2_, *ω*_3_) to project the selected genes onto correlation circles. Figures 5 and 6 provide an illustrative example of these types of figures in the case of sPLS. These graphical outputs proposed by [31] improve the interpretability of the results in the following manner. First they allow for the identification of correlated gene subsets from each data set, *i.e*. with similar expression profiles. Second they help revealing the correlations between gene subsets from both data sets (by superimposing both graphics). And third they help relating these correlated subsets to the associated tumor cell lines by combining the information contained in Figures 5, 6 and Figure 4(a). For example, the genes that were selected on the second sPLS dimension for both data sets should help discriminating melanoma tumors from the other cell lines.

If the loading vectors are orthogonal (*i.e*. if *cor*(*a*_*s*_, *a*_*r*_) = 0, *cor*(*b*_*s*_, *b*_*r*_) = 0, *r *<*s*), there is a small degree of overlap between the genes selected in each dimension (Table 2). In this case, this means that each selection focuses on a specific aspect of the data set, for example a specific tumor type. This valuable orthogonal property between loading vectors is kept in the sparse methods (sPLS, CCA-EN), which is not often the case, for example with the sparse PCA approaches [8,23,32]. The gene lists selected with CCA-EN and sPLS are hence almost not redundant across all dimensions. In fact, only 0 to 2 genes are overlapping between dimensions 1–2 and 1–3 in the Ross data set, and between 1 to 13 genes in the Staunton data set for both approaches (Table 2). On the contrary, there is no orthogonality between CIA loadings vectors, leading to a high number of overlapping genes (up to 31 between dimensions 1 and 2).

**Table 2:** Comparisons between gene lists.

	X: dim 1–2	dim 1–3	dim 2–3	dim 1–2–3	Y: dim 1–2	dim 1–3	dim 2–3	dim 1–2–3
CCA-EN	0	2	2	0	1	3	13	1
CIA	20	17	31	2	14	21	24	1
sPLS	0	0	2	0	0	8	1	0

Number of genes commonly selected (overlap) between all dimensions for each approach for X = Ross-cDNA data set (left-hand side of the table) and for Y = Staunton-Affymetrix data set (right-hand side of the table).

#### Analysis of the gene lists

Based on the interpretation of the cell line clusters, we analysed three sets of gene lists (3 methods × 2 data sets = 6 lists of 100 genes per set, see Additional files 2, 3, 4 for each heat map of each gene list):

-**Set 1: **the lists associated with the separation of cell lines with epithelial (mainly LE and CO) *vs*. mesenchymal (mainly RE and CNS) characteristics (CCA-EN dimension 2, CIA and sPLS dimension 1),

-**Set 2: **the lists associated with the separation of the melanoma cell lines (ME, BR_MDAN and BR_MDAMB435) from the other cell lines (CCA-EN dimension 1, CIA and sPLS dimension 2),

-**Set 3: **the lists associated with the separation of the LE cell lines from the CO cell lines (dimension 3 for each method, see Figures 3(b) and 4(b)).

**Figure 7:**
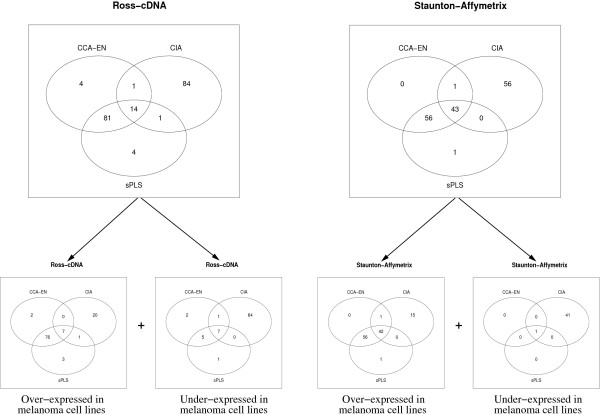
**Venn Diagrams**. Venn diagrams for 100 selected genes associated to melanoma *vs*. the other cell lines for each data set (top). These lists were then decomposed into up and down regulated genes (bottom).

For each set of gene lists we evaluated the number of genes that were commonly selected by the different methods. For example, figure 7 displays the Venn diagrams for the lists of genes characterizing the melanoma cell lines (Set 2). These Venn diagrams revealed a very strong similarity between the CCA-EN and sPLS gene lists, whereas CIA selected different genes characterizing the cell lines. Similar results were obtained for Set 1 and Set 3 and the same trend was observed when more than 100 variables were selected on each dimension (data not shown).

For each dimension and each method, we evaluated the overlap between the gene lists obtained from the two initial data sets. We would expect from such canonical correlation-based methods that they identify high correlations between features selected from the two platforms, when these features actually measure the expression of the same gene. To evaluate this aspect, the identifiers of the features from each platform were mapped to unique gene identifiers using Ingenuity Pathways Analysis application (IPA, ). For each dimension, CCA-EN and sPLS selected approximately 20 features from the Ross and Staunton data sets that corresponded to identical genes. In contrast, CIA selected 15 to 17 identical genes between the two data sets. The heatmaps for each of the 18 gene lists (Additional files 2, 3, 4) illustrated well the general finding that CCA-EN and sPLS yield highly similar lists of genes exhibiting expression patterns which characterize well the cell lines separated along each dimension. In contrast, CIA tends to select genes with a higher variance across all cell lines compared to CCA-EN and sPLS.

#### Analysis of the gene lists with IPA

Finally, we evaluated the biological relevance of the genes selected by each method. The 3 sets of gene lists were loaded into IPA along with their corresponding log ratios (*i.e*. Set 1: mean expression in LE+CO/mean expression in RE+CNS, Set 2: mean expression in ME+BR MDAN+BR MDAMB435/mean expression in the other cell lines, Set 3: mean expression in LE/mean expression in CO). We focused on:

1) *biological functions *that were significantly over-represented (right-tailed Fisher's exact test) in the gene lists compared to the initial data sets,

2) *canonical pathways *in which the selected genes were significantly over-represented compared to the genes in the initial data sets and

3) the first *networks *generated by IPA from the gene selections. These networks are built by combining the genes into small networks (35 molecules maximum) that maximize their specific connectivity [33]. This results in highly-interconnected networks.

##### Over-represented biological functions

For the three methods, the over-represented biological functions in the different gene lists were generally relevant to the cell lines separated along each corresponding dimensions. The epithelial to mesenchymal transition (EMT, Set 1), a key process for tissue remodelling during embryonic development, could contribute to establish the metastatic potential of carcinoma cells [34]. Studying the events underlying the EMT is thus of primary importance to better understand tumor malignancy. During the EMT, cells acquire morphological and biochemical characteristics that enables them to limit their contacts with neighbouring cells and to invade the extracellular matrix. Accordingly, for Set 1, the three methods identified biological functions related to cellular movement, connective tissue development and cell-to-cell signalling and interaction (see Additional files 5 and 6) which directly relate to the EMT. Melanomas (Set 2) originate from skin melanocytes which are pigment cells producing melanin, the synthesis of which involves the amino acids tyrosine and cysteine. Accordingly, for Set 2, the different methods identified biological functions related to skin biology and to amino acid metabolism (not shown). Finally, LE cell lines represent leukaemia which result from the abnormal proliferation of blood cells while CO cell lines represent colon carcinomas which originate from epithelial cells of the colon (Set 3). Not surprisingly, the different methods identified lists of genes linked to the functions and diseases of the haematological and immunological systems which were differentially expressed between LE and CO cell lines (not shown).

The three methods extracted complementary findings from the two data sets. Particularly, they frequently identified similar biological functions supported by different genes from the two platforms.

One major finding from this analysis was that CIA identified many more significant biological functions compared to CCA-EN/sPLS. For example for the Ross/Set 1 data, CCA-EN and sPLS identified 7 functions with p < 0:001 while CIA identified 21 different functions using the same threshold. However, the functions identified by CIA were highly redundant between the three sets, as a result of important overlaps in the gene lists selected by this method (Table 2). Additionally, CIA recurrently identified categories representing relatively general functions for tumor cells such as cell death, cancer or cell morphology. Overall, the findings obtained by CCA-EN and sPLS were much more specific and allowed a deeper understanding of the biological processes characterizing the different cell lines.

##### Canonical pathways

In accordance with this observation CCA-EN and sPLS generally found more significant canonical pathways compared to CIA. This likely results from redundant and less specific genes contained in the CIA gene selections, hence limiting the enrichment of a sufficient number of genes in a given pathway. In particular, the integrin and actin sytoskeleton pathways were only identified by CCA-EN and sPLS for Set 1. These two pathways are central to cellular movement and for the interactions with the extracellular matrix. Consistently, several genes from these pathways, including integrins *α *and *β*, caveolin, *α*-actinin and vinculin are over-expressed in RE and CNS cell lines (mesenchymal) compared to LE and CO cell lines (epithelial). For Set 2, all three methods identified the overexpression of genes from the tyrosine metabolism pathway in melanoma cell lines, in particular tyrosinase, tyrosinase related proteins 1 and 2 and dopachrome tautoisomerase which are involved in melanin synthesis. However, only CCA-EN and sPLS identified glycosphingolipid (ganglioside and globosid) biosynthesis pathways as characterizing the melanoma cell lines. Melanoma tumors are known to be rich in these glycosphingolipids [35]. Indeed, their presence at the cell membrane makes them interesting targets for immunotherapy and vaccination strategies [30]. Among the pathways identified for Set 3, only sPLS identified the tight junction signalling pathway (in particular Claudin 4 and Zona occludens 1) as characterizing CO cell lines compared to LE cell lines. This is consistent with the typical epithelial characteristics of the CO cell lines.

##### Networks

We explored the networks generated by IPA from each gene list. For Set 1, the first networks for each method were highly connected and were mainly related to cellular movement. Interestingly, all networks pointed to the extracellular signal-regulated kinase (ERK) as a central player in the expression of the selected genes, which is consistent with the role of the ERK pathway in cell migration [36]. When we merged the first networks obtained from the three methods, highly similar networks were obtained for the two platforms (Additional files 7 and 8) but only the Staunton data set highlighted the transforming growth factor-*β *(TGF-*β*) pathway, which is thought to be a primary inducer of the EMT [34]. Despite this difference, the most connected nodes (including integrins *α *and *β*, *α*-actinin, connective tissue growth factor, fibronectin 1, SERPINE1, plasminogen activator urokinase, Ras or ERK) were found in both networks. These likely represent central players in establishing the different phenotypes of LE and CO cell lines on one hand and of RE and CNS cell lines on the other hand. The networks characterizing melanoma cell lines (Set 2, not shown) highlighted several markers used for the diagnosis of melanomas including the over expressed MITF, vimentin, S-100A1, S-100B and Melan-A and the under expressed keratins 7, 8, 18 and 19. Finally, the networks generated for Set 3 highlighted many genes involved in cell-cell contacts, cell adhesion and cellular movement which were generally expressed at higher levels in CO compared to LE cell lines.

## Conclusion

The analysis of the NCI60 data sets with CCA-EN, CIA and sPLS evidenced the main differences between these methods.

### CIA

CIA does not propose a built-in variable selection procedure and requires a two-step analysis to perform variable selection. The main individual effects were identified. However, the loadings or weight vectors obtained were not orthogonal, in contrary to CCA-EN and sPLS. This resulted in some redundancy in the gene selections, which may be a limitation for the biological interpretation, as it led to less specific results.

### CCA-EN

CCA-EN first captured the main robust effect on the individuals that was present in the two data sets. As a consequence, it may hide strongest individual effects that are present in only one data set. We observed a strong similarity between CCA-EN and sPLS in the gene selections, except that the first two axes were permuted. In fact, we believe that CCA-EN can be considered as a sparse PLS variant with a canonical mode. Indeed, the elastic net is approximated with a univariate threshold, which is similar to a lasso soft-thresholding penalization, and the whole algorithm uses PLS and not CCA computations. This explains why the canonical correlations do not monotonically decrease. The only difference that distinguishes sPLS canonical mode from CCA-EN is the initialization of the algorithm for each dimension. CCA-EN maximizes the correlation between the latent variables, whereas sPLS maximizes the covariance.

### sPLS

We found that sPLS made a good compromise between all these approaches. It includes variable selection and the loading vectors are orthogonal. Although sPLS and CCA-EN do not order the axis in the same manner, both approaches were highly similar, except for slight but significant differences when studying LE *vs*. CO (Set 3). In this particular case, the resulting gene lists clearly provided complementary information.

Based on the present study, we would primarily recommend the use of CCA-EN or sPLS when gene selection is an issue. Like CCA-EN, sPLS includes a built-in variable selection procedure but captured subtle individual effects. Therefore, these two approaches may differ when computing the fist axes. All approaches are easy to use and fast to compute. These approaches would benefit from the development of an R package to harmonize their inputs and outputs so as to facilitate their use and their comparison.

## Authors' contributions

KALC developed the algorithm, performed the statistical analyses. PGPM performed the biological interpretation. KALC and PGPM wrote the manuscript, CRG, PGPM and PB participated in the design of the study. All authors read and approved the final manuscript.

## Supplementary Material

Additional File 1 **Algorithms.** The algorithms PLS, sPLS and CCA-EN are detailed. Click here for file

Additional File 2 **Hierarchical clusterings, epithelial *vs*. mesenchymal-like (Set 1).** Heat map displays of hierarchical clustering results with the Ward method and correlation distance with genes in lines and cell lines in columns. Samples are clustered according to the dendrograms obtained in Figure 2. The red (green) colour represents over-expressed (under-expressed) genes. Genes from Set 1 are displayed for each method. Click here for file

Additional File 3 **Hierarchical clusterings, melanoma (Set 2). **Heat map displays of hierarchical clustering results with the Ward method and correlation distance with genes in lines and cell lines in columns. Samples are clustered according to the dendrograms obtained in Figure 2. The red (green) colour represents over-expressed (under-expressed) genes. Genes from Set 2 are displayed for each method. Click here for file

Additional File 4 **Hierarchical clusterings, LE *vs*. CO cell lines (Set 3).** Heat map displays of hierarchical clustering results with the Ward method and correlation distance with genes in lines and cell lines in columns. Samples are clustered according to the dendrograms obtained in Figure 2. The red (green) colour represents over-expressed (under-expressed) genes. Genes from Set 3 are displayed for each method. Click here for file

Additional File 5 **Biological functions from Set 1 for the Ross data set.** Biological functions significantly over-represented in the gene lists selected from the Ross data set by the three methods CCA-EN, CIA and sPLS (Set 1 of gene lists). Only the biological functions with a p-value lower than 0.001 for all three methods are presented. "x" indicates how the genes were selected. The analysis was performed using Ingenuity Pathways Analysis application  which evaluates the over-representation of functional categories through a right-tailed Fisher's exact test. Click here for file

Additional File 6 **Biological functions from Set 1 for the Staunton data set.** Biological functions significantly over-represented in the gene lists selected from the Staunton data set by the three methods CCA-EN, CIA and sPLS (Set 1 of gene lists). Only the biological functions with a p-value lower than 0.001 for all three methods are presented. "x" indicates how the genes were selected. The analysis was performed using Ingenuity Pathways Analysis application  which evaluates the over-representation of functional categories through a right-tailed Fisher's exact test. Click here for file

Additional File 7 **Network from the Ross gene list, Set 1.** Molecular network obtained from the Ross gene lists from Set 1. For each canonical method (CCA-EN, CIA or sPLS), molecular networks were built from the Ross gene lists (focus genes) of Set 1 using Ingenuity Pathways Analysis (IPA, ). The first networks obtained from each method were merged into the presented network. Green and red colors indicate under- and over-expressions respectively in the LE/CO cell lines compared to the RE/CNS cell lines for the genes that were selected by sPLS. Genes that were selected by CCA-EN or CIA are in grey and were all under-expressed in the LE/CO cell lines compared to the RE/CNS cell lines. Genes in white have been added by IPA based on their high connectivity with focus genes. Click here for file

Additional File 8 **Network from the Staunton gene list, Set 1.** Molecular network obtained from the Staunton gene lists from Set 1. For each canonical method (CCA-EN, CIA or sPLS), molecular networks were built from the Staunton gene lists (focus genes) of Set 1 using Ingenuity Pathways Analysis (IPA, ). The first networks obtained from each method were merged into the presented network. Green and red colors indicate under- and over-expressions respectively in the LE/CO cell lines compared to the RE/CNS cell lines for the genes that were selected by sPLS are colored in red or green. Genes that were selected by CCA-EN or CIA are in grey and were all under-expressed in the LE/CO cell lines compared to the RE/CNS cell lines. Genes in white have been added by IPA based on their high connectivity with focus genes. Click here for file
