# Supplementary material for: Sparse canonical methods for biological data integration: application to a cross-platform study
Source: BMC Bioinformatics. 2009 Jan 26;10:34. doi: 10.1186/1471-2105-10-34 (PMC2640358; doi:10.1186/1471-2105-10-34)
Supplement: Additional File 1 — Algorithms. The algorithms PLS, sPLS and CCA-EN are detailed. [file 1471-2105-10-34-S1.pdf]

1 PLS algorithm (canonical mode)

1. $X_0 = X$, $Y_0 = Y$

2. For $h = 1 \dots H$:

   (a) Initialize
   ξ_h = first column of X_{h-1} $\quad \omega_h$ = first column of Y_{h-1}

   (b) Until convergence of a_h:

      i. $a_h = X'_{h-1}\xi_h/\xi'_h\xi_h$, norm a_h

      ii. $\xi_h = X_{h-1}a_h$, norm ξ_h

      iii. $b_h = Y'_{h-1}\xi_h/\xi'_h\xi_h$, norm b_h

      iv. $\omega_h = Y_{h-1}b_h$, norm ω_h

   (c) $c_h = X'_{h-1}\xi_h \qquad e_h = Y'_{h-1}\omega_h$

   (d) $X_h = X_{h-1} - \xi_h c'_h \quad Y_h = Y_{h-1} - \omega_h e'_h$

Step (c) computes the regression coefficients of the matrices X_{h-1} and Y_{h-1} on the latent variables ξ_h and ω_h.
Step (d) computes the deflated (residual) matrices.

2 sparse PLS algorithm (canonical mode)

Sparse PLS initializes step (a) in PLS by extracting the first pair of singular vectors (a_h, b_h) of the crossproduct $X'_{h-1}Y_{h-1}$, which includes variation in both X and Y and the correlation between the two sets.
The two loading vectors (a_h, b_h) are then computed with penalizations λ_a and λ_b in step (b), and the latent vectors (ξ_h, ω_h) are then computed, where $g_\lambda(y) = sign(y)(|y| - \lambda)_+$ is the soft-thresholding penalty function.

1. $X_0 = X \qquad Y_0 = Y$

2. For $h = 1 \dots H$:

   (a) Set $\tilde{M}_{h-1} = X'_{h-1}Y_{h-1}$, decompose $\tilde{M}_{h-1}$ and extract the first pair of singular vectors $a_{old} = a_h$ and $b_{old} = b_h$

   (b) Until convergence of a_{new} and b_{new}:

      i. $a_{new} = g_{\lambda_a}(\tilde{M}_{h-1}b_{old})$, norm a_{new}

      ii. $b_{new} = g_{\lambda_b}(\tilde{M}'_{h-1}a_{old})$, norm b_{new}

      iii. $a_{old} = a_{new}$, $b_{old} = b_{new}$

   (c) $\xi_h = X_{h-1}a_{new}$
   $\omega_h = Y_{h-1}b_{new}$

   (d) $c_h = X'_{h-1}\xi_h \quad e_h = Y'_{h-1}\omega_h$

   (e) $X_h = X_{h-1} - \xi_h c'_h \quad Y_h = Y_{h-1} - \omega_h e'_h$

3 Canonical Correlation Analysis with Elastic Net penalization

CCA-EN initializes step (a) in PLS by setting $\xi_h = X^j_{h-1}$ and $\omega_h = Y^k_{h-1}$ such that $cor(X^j_{h-1}, Y^k_{h-1})$ is maximized, for $j = 1 \dots p$ and $k = 1 \dots q$. Hence, this algorithm aims at maximizing the correlation (rather than the covariance for PLS and sPLS).
The approximation on Elastic Net penalization finally consists in introducing soft-thresholding penalizations, as in sparse PLS, which makes both algorithms similar, except for the initilization step.

1. $X_0 = X \qquad Y_0 = Y$
2. For $h = 1 \dots H$:
   (a) Set $\xi_h = X^j_{h-1}$ and $\omega_h = Y^k_{h-1}$ such that $cor(X^j_{h-1}, Y^k_{h-1})$ is maximized
   $a_{new} = X'_{h-1}\xi_h/\xi'_h\xi_h \quad b_{new} = Y'_{h-1}\xi_h/\xi'_h\xi_h$, norm a_{new} and b_{new}
   (b) Until convergence of a_{new} and b_{new}:
      i. $a_{new} = g_{\lambda_a}(Y_{h-1}b_{old})$, norm a_{new}
      ii. $b_{new} = g_{\lambda_b}(X_{h-1}a_{old})$, norm b_{new}
      iii. $a_{old} = a_{new}$, $b_{old} = b_{new}$
   (c) $\xi_h = X_{h-1}a_{new}$, norm ξ_h
   $\omega_h = Y_{h-1}b_{new}$ norm ω_h
   (d) $c_h = X'_{h-1}\xi_h \quad e_h = Y'_{h-1}\omega_h$
   (e) $X_h = X_{h-1} - \xi_h c'_h \quad Y_h = Y_{h-1} - \omega_h e'_h$
