# Supplementary figures and images for: Sparse canonical methods for biological data integration: application to a cross-platform study
Source: BMC Bioinformatics. 2009 Jan 26;10:34. doi: 10.1186/1471-2105-10-34 (PMC2640358; doi:10.1186/1471-2105-10-34)

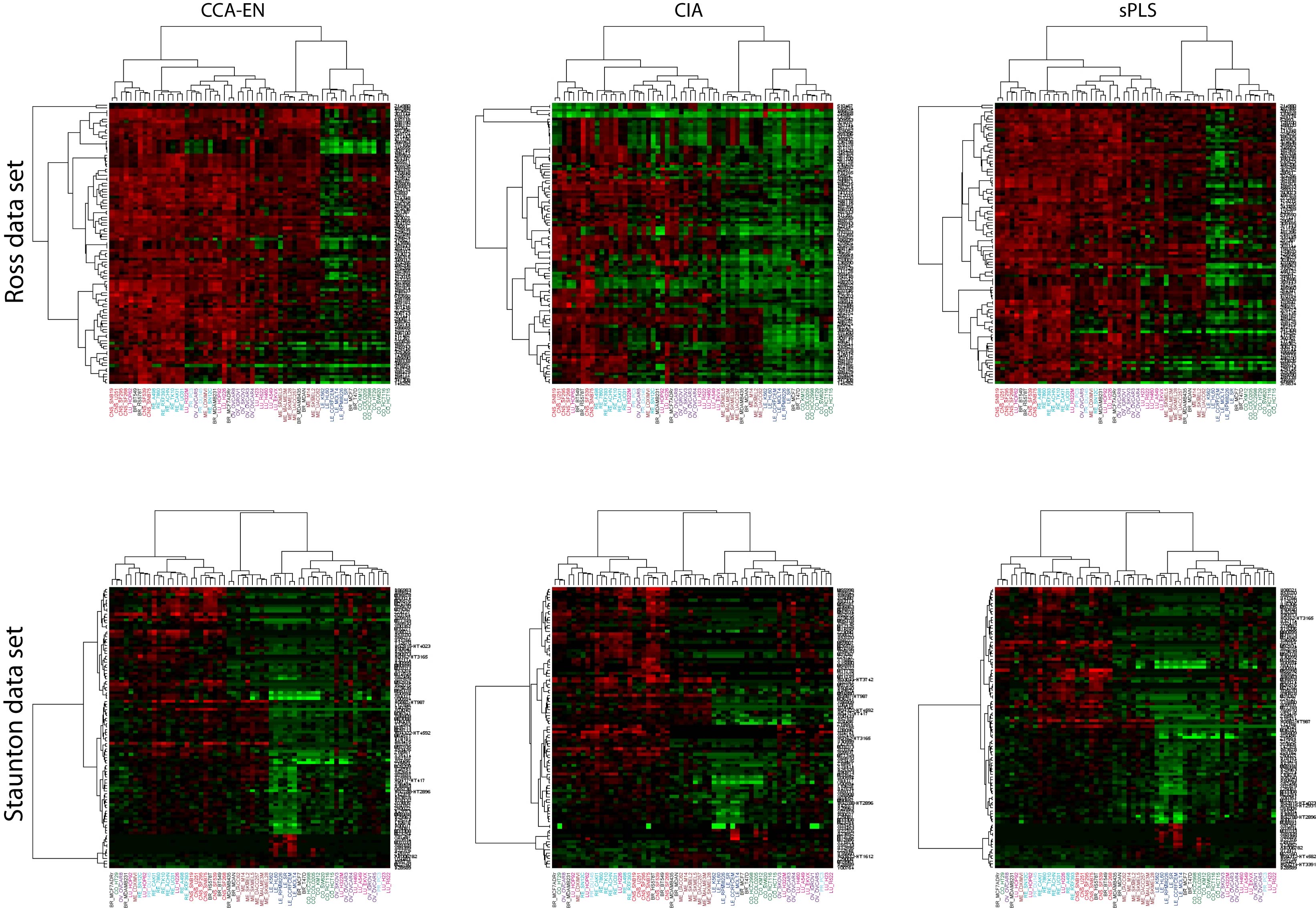

Supplement: Additional File 2 — Hierarchical clusterings, epithelial vs. mesenchymal-like (Set 1). Heat map displays of hierarchical clustering results with the Ward method and correlation distance with genes in lines and cell lines in columns. Samples are clustered according to the dendrograms obtained in Figure 2. The red (green) colour represents over-expressed (under-expressed) genes. Genes from Set 1 are displayed for each method. [file 1471-2105-10-34-S2.jpeg]

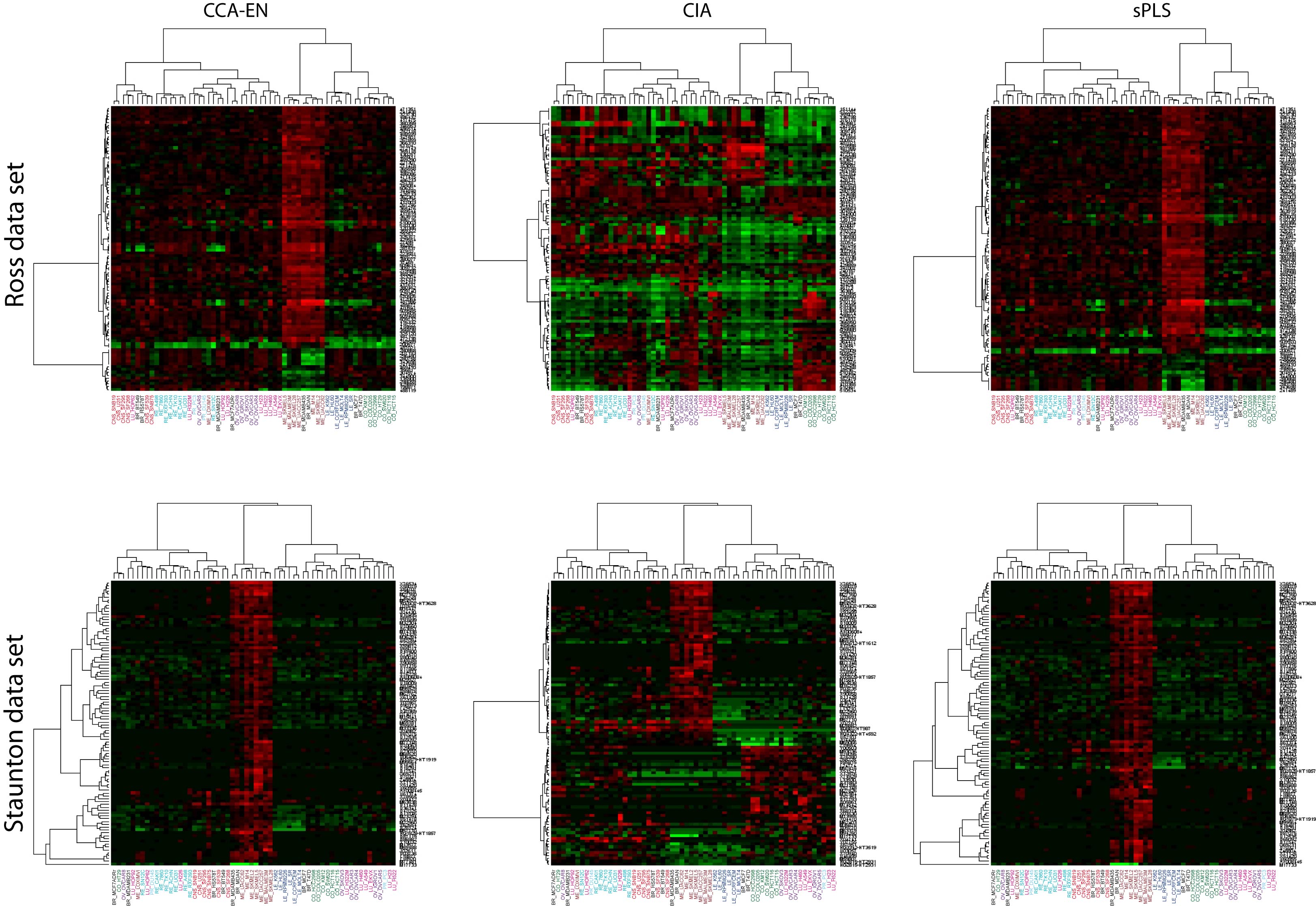

Supplement: Additional File 3 — Hierarchical clusterings, melanoma (Set 2). Heat map displays of hierarchical clustering results with the Ward method and correlation distance with genes in lines and cell lines in columns. Samples are clustered according to the dendrograms obtained in Figure 2. The red (green) colour represents over-expressed (under-expressed) genes. Genes from Set 2 are displayed for each method. [file 1471-2105-10-34-S3.jpeg]

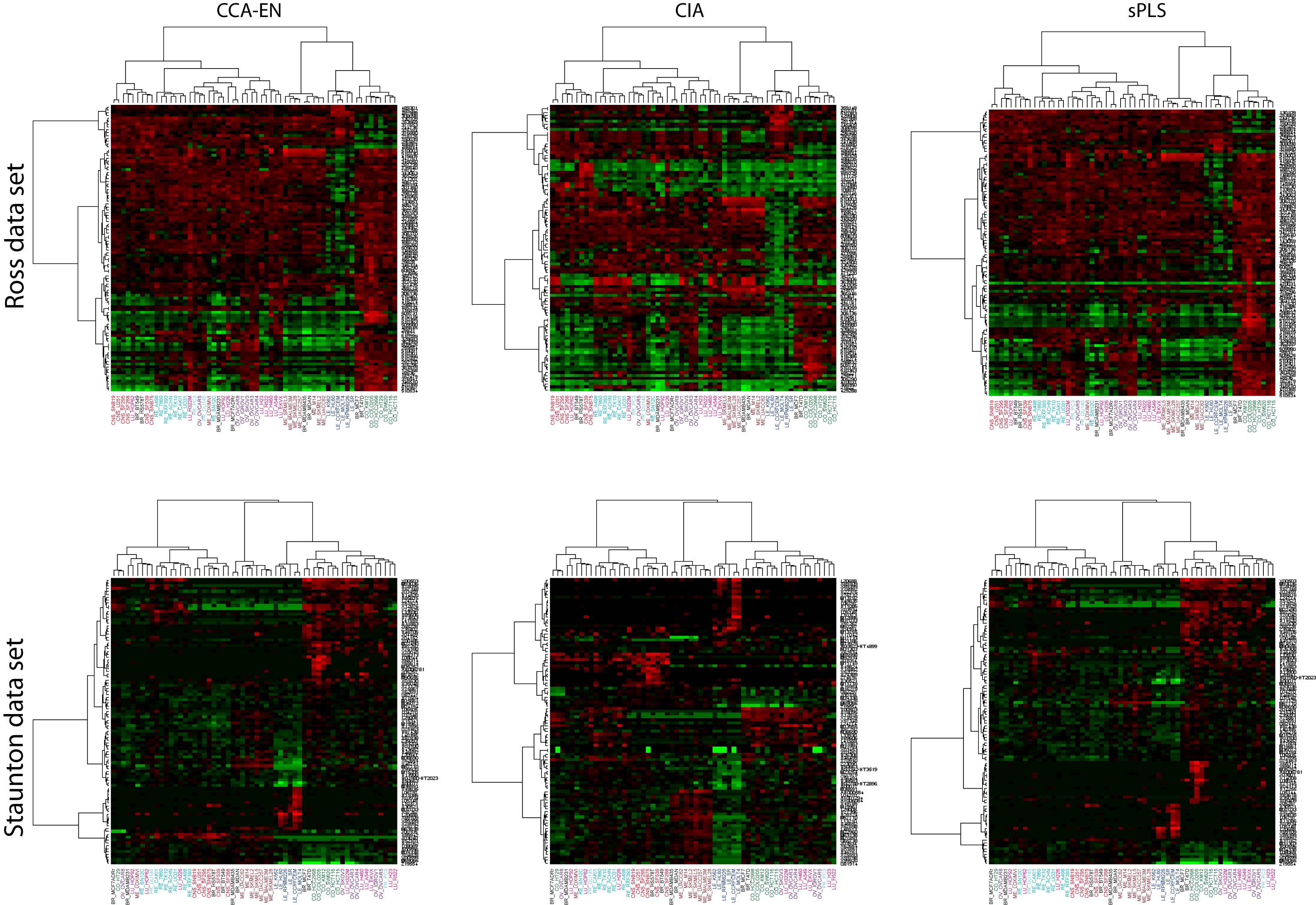

Supplement: Additional File 4 — Hierarchical clusterings, LE vs. CO cell lines (Set 3). Heat map displays of hierarchical clustering results with the Ward method and correlation distance with genes in lines and cell lines in columns. Samples are clustered according to the dendrograms obtained in Figure 2. The red (green) colour represents over-expressed (under-expressed) genes. Genes from Set 3 are displayed for each method. [file 1471-2105-10-34-S4.jpeg]
